# Supplementary material for: A Global Screen for Assembly State Changes of the Mitotic Proteome by SEC-SWATH-MS
Source: Cell Syst. 2020 Feb 26;10(2):133–155.e6. doi: 10.1016/j.cels.2020.01.001 (PMC7042714; doi:10.1016/j.cels.2020.01.001)

Q8WVX9 | FACR1_HUMAN | FAR1 MLSTD2 UNQ2423/PRO4981
Monomer MW [kDa]: 59.357 Monomer expected elution fraction: 45

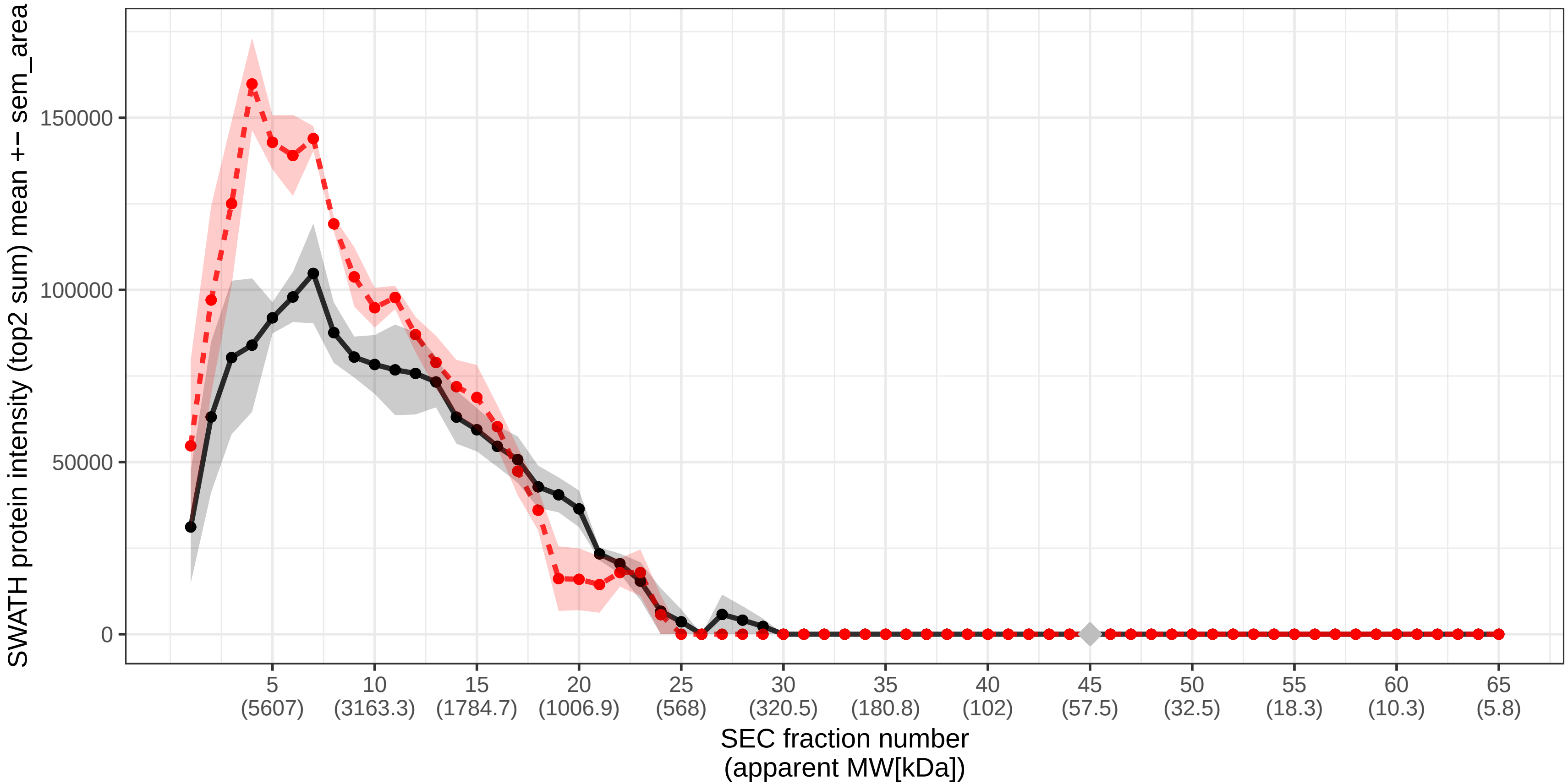

Supplement: Data S1. SEC-SWATH-MS Protein Chromatograms, Related to Figure 1 [file mmc6.zip › SECchrom_Q8WVX9_FACR1_HUMAN_FAR1_MLSTD2_UNQ2423_PRO4981.pdf]
